# Leveraging Hypotension Prediction Index to Forecast LPS-Induced Acute Lung Injury and Inflammation in a Porcine Model: Exploring the Role of Hypoxia-Inducible Factor in Circulatory Shock

**DOI:** 10.3390/biomedicines12081665

**Published:** 2024-07-25

**Authors:** Yuan-Ming Tsai, Yu-Chieh Lin, Chih-Yuan Chen, Hung-Che Chien, Hung Chang, Ming-Hsien Chiang

**Affiliations:** 1 Division of Thoracic Surgery, Department of Surgery, Tri-Service General Hospital, National Defense Medical Center, Taipei 114201, Taiwan; hung@mail.ndmctsgh.edu.tw; 2 Department of Physiology and Biophysics, National Defense Medical Center, Taipei 114201, Taiwan; information924@gmail.com (C.-Y.C.); chienhc@mail.ndmctsgh.edu.tw (H.-C.C.); 3 Department of Pathology and Laboratory Medicine, Taoyuan Armed Forces General Hospital, Taoyuan 325208, Taiwan; medicalwolf1981@gmail.com; 4 Department and Graduate Institute of Biology and Anatomy, National Defense Medical Center, Taipei 114201, Taiwan; 5 Department of Nutritional Science, College of Human Ecology, Fu Jen Catholic University, New Taipei City 242062, Taiwan

**Keywords:** hemodynamic monitoring, hypotension prediction index, lipopolysaccharide, lung injury, hypotension, hypoxia-inducible factor

## Abstract

Acute respiratory distress syndrome (ARDS) is a critical illness in critically unwell patients, characterized by refractory hypoxemia and shock. This study evaluates an early detection tool and investigates the relationship between hypoxia and circulatory shock in ARDS, to improve diagnostic precision and therapy customization. We used a porcine model, inducing ARDS with mechanical ventilation and intratracheal plus intravenous lipopolysaccharide (LPS) injection. Hemodynamic changes were monitored using an Acumen IQ sensor and a ForeSight Elite sensor connected to the HemoSphere platform. We evaluated tissue damage, inflammatory response, and hypoxia-inducible factor (HIF) alterations using enzyme-linked immunosorbent assay and immunohistochemistry. The results showed severe hypotension and increased heart rates post-LPS exposure, with a notable rise in the hypotension prediction index (HPI) during acute lung injury (*p* = 0.024). Tissue oxygen saturation dropped considerably in the right brain region. Interestingly, post-injury HIF-2α levels were lower at the end of the experiment. Our findings imply that the HPI can effectively predict ARDS-related hypotension. HIF expression levels may serve as possible markers of rapid ARDS progression. Further research should be conducted on the clinical value of this novel approach in critical care, as well as the relationship between the HIF pathway and ARDS-associated hypotension.

## 1. Introduction

Acute respiratory distress syndrome (ARDS) represents the most severe form of acute lung injury, with a high mortality rate of up to 40% in critically ill patients [1]. This high mortality is mostly caused by abrupt hypoxemic respiratory failure and lung inflammation. ARDS is caused by pulmonary or systemic inflammation, leading to cytokine release and other proinflammatory molecules that increase lung permeability and reduce cardiac output, thereby precipitating cardiovascular complications [2,3]. Diagnostic criteria for ARDS include a PaO_2_:FiO_2_ ratio of less than 300 mmHg and bilateral opacification apparent on chest imaging at a positive end-expiratory pressure (PEEP) of 5 cm H_2_O within 7 days of an injury [4]. Despite varied degrees of severity, ARDS is characterized by decreased lung compliance, impaired gas exchange, and severe hypoxemia. The alveolar–capillary barrier is extensively damaged, resulting in hypoxia, and endothelial and epithelial cell death [5,6]. Lipopolysaccharide (LPS)-induced acute lung damage is distinguished by significant lung edema, breakdown of endothelial and epithelial barriers, widespread neutrophil infiltration, and the release of inflammatory mediators. Previous research has shown that LPS plays a vital role in causing acute respiratory epithelial damage during sepsis [7].

Shock is frequently associated with ARDS as a result of pulmonary circulation malfunction, which can lead to cardiac issues later in the disease progression. This can lead to right ventricular (RV) failure, which is a key vulnerability in ARDS, with the most severe form being acute cor pulmonale [8]. The increased risk of cardiovascular disease highlights the need for meticulous monitoring of intravascular volume status and the adoption of conservative fluid management in ARDS treatment [9]. However, precisely estimating intravascular volume is difficult, which can delay the detection of end-organ hypoperfusion. The FloTrac system, a minimally invasive device for arterial pressure-based cardiac output monitoring, has been called into doubt in septic patients with low systemic vascular resistance (SVR), a common characteristic in ICU patients [10,11]. Furthermore, the stroke volume variation (SVV) produced by FloTrac may not provide reliable information for fluid optimization in such cases [12]. Consequently, it is critical to investigate innovative monitoring for managing sepsis-induced ALI.

The brain’s function and viability are highly dependent on an appropriate oxygen supply. Reduced blood pressure can cause cerebral desaturation and other neurological complications. Direct assessment of regional cerebral oxygen saturation using near-infrared spectroscopy, which differs from pulse oximetry in that it averages oxygenation across the arterial, capillary, and venous flow of the underlying tissue, aids in the early detection and treatment of such issues. While criteria for cerebral desaturation are not universally accepted, they frequently include reductions of more than 20% from baseline or an absolute value of less than 50%, with some guidelines recommending early intervention for reductions greater than 10% [13].

Pulmonary hypoxia is a hallmark of several inflammatory lung diseases, particularly ARDS, where despite advances in supportive care, mortality rates remain alarmingly high [14]. Hypoxia-inducible transcription factors (HIFs, HIF-1α, and HIF-2α) are crucial for vascular adaptation and cellular response to hypoxia. These factors are not only involved in ARDS but also play important roles in various physiological and pathological processes associated with cardiovascular diseases [15,16]. The role of the HIF-α signaling pathway in ARDS is of special interest. The pathway’s activation during hypoxic conditions can exacerbate hypoxia-induced damage via various mechanisms, including inflammation and metabolic process changes. The HIF-α pathway presents a complex but promising therapeutic target due to its dual nature. Our research focuses on a novel monitoring sensor that can accurately predict arterial hypotension and cerebral hypoxia. Our goal is to elucidate the intricate interactions between HIF-α signaling and circulatory shock to better understand how the HIF-α pathway’s response to hypoxia contributes to circulatory shock in ARDS. This research is critical because it informs the development of targeted interventions that can reduce the serious complications associated with ARDS-induced hypoxia and shock.

## 2. Materials and Methods

The animal experiment procedures were designed to reduce animal pain, resulting in only mild to moderate discomfort. This study was approved by the Animal Care and Use Committee of the National Defense Medical Center (Approval No.: IACUC-21-072).

### 2.1. Animal Preparation

The 10–12-week-old male pigs weighing 20–30 kg were premedicated intramuscularly with tiletamine–zolazepam at a dose of 5 mg/kg and atropine at a dose of 0.05 mg/kg prior to anesthesia. Anesthesia and neuromuscular blockade were induced using pentobarbital sodium (8–12 mg/kg/h), fentanyl (20–30 mg/kg/h), and cisatracurium (0.1 mg/kg/h). All pigs will have continuous invasive arterial pressure monitoring via an indwelling left femoral arterial catheter connected to an Acumen IQ sensor and a pair of ForeSight Elite sensors linked to the HemoSphere monitor platform (Edwards Lifesciences, Irvine, CA, USA).

### 2.2. Induction of ARDS by Lipopolysaccharides

Following anesthesia, the pigs were positioned supine on the operating bed. The pigs underwent endotracheal tube insertion via tracheostomy and mechanical ventilation in volume-controlled mode with a tidal volume of 6 mL/kg body weight, an inspired oxygen fraction of 50%, and a PEEP of 5 cm H_2_O. Following injury induction, the tidal volume was set to 8 mL/kg, and the breathing rate was adjusted to keep the end-tidal CO_2_ pressure (EtCO_2_) between 35 to 45 mmHg [17]. Pulmonary injury was performed by intratracheal administration of 0.33 mg/kg lipopolysaccharides (LPS) and continuous infusion of 0.2 mL/kg of LPS from gram-negative bacteria *Escherichia coli* (O111:B4, Sigma-Aldrich, Merck, Darmstadt, Germany) via an infusion pump for 20 min through the right internal jugular vein.

### 2.3. Hemodynamic Data Collection

**Figure 1:**
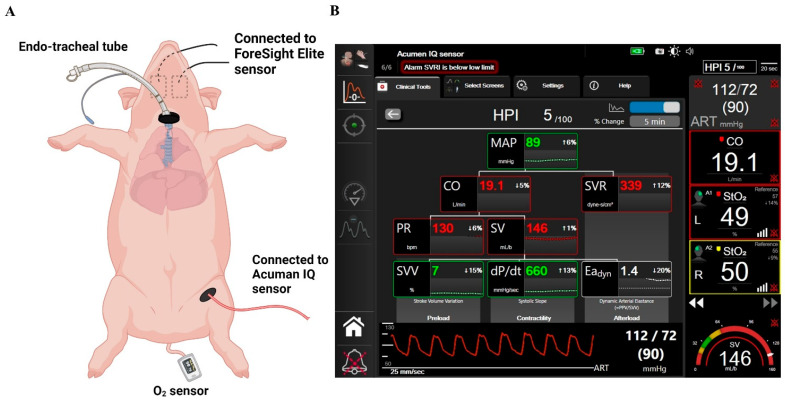
Experimental overview. (**A**) Schematic of ARDS induction: lung injury induced by intratracheal delivery of LPS to bilateral lungs, followed by continuous infusion of LPS through the right internal jugular vein. (**B**) The screen of the HPI and tissue oxygen saturation algorithm. Abbreviations: ARDS, acute respiratory distress syndrome; LPS, lipopolysaccharide; HPI, hypotension prediction index.

During the experimental procedure, the following time points were recorded: baseline, after LPS administration (injury), and 1, 2, and 3 h after ARDS. The severity of ARDS is classified based on the ratio of partial pressure of oxygen in arterial blood (PaO_2_) to the fraction of inspiratory oxygen concentration (FiO_2_). At each time point, arterial systolic blood pressure (mmHg), diastolic blood pressure (mmHg), mean arterial pressure (MAP, mmHg), heart rate (HR, beats per minute), cardiac output (CO, L/min), stroke volume (SV, mL/beat), stroke volume variation (SVV, %), systemic vascular resistance (SVR, dyn·s·cm^−5^/m^2^), systolic pressure increase divided by duration of systole as an indicator of left ventricular contractility (dP/dt, mmHg/sec), dynamic arterial elastance (Ea_dyn_, expressed in arbitrary units), hypotension prediction index (HPI, %), and tissue oxygen saturation (StO_2_, %) were measured by the system and recorded from the screen monitor (Figure 1).

### 2.4. Measurement of Cytokine and Hypoxia-Inducible Factor

Blood samples were taken at baseline, after LPS-induced lung injury, and at 1, 2, and 3 h after ARDS onset. Inflammatory cytokines (IL1-β, IL-6, IL-8, and TNF-α) were measured using enzyme-linked immunosorbent assay (ELISA) kits (Catalog #MEK1018, Boster Bio, Pleasanton, CA, USA). Cytokine levels were determined using the manufacturer’s instructions. Specifically, porcine HIF-1α and HIF-2α levels were determined using ELISA kits supplied by MyBioSource (San Diego, CA, USA) (Catalog #MBS2503432 for HIF-1α and MBS2600613 for HIF-2α).

### 2.5. Gas Chromatography–Mass Spectrometry (GC-MS) Analysis

Blood samples were gently thawed at 4 °C. Each sample (500 μL) was mixed with 1 mg/mL tropine acid as an internal standard. This mixture was treated with a hydrochloric acid hydrazine solution and a 2.5 mol/L NaOH solution, vortexed for 10 min, and then allowed to react at room temperature for 30 min. After adding 6 mol/L hydrochloric acid and 5 mL of ethyl acetate, the mixture was vigorously vortexed for another 10 min before centrifugation for 5 min. The supernatant was evaporated using a gentle stream of nitrogen at room temperature. To derivatize, 100 μL of BSTFA-TMCS reagent was added, and the mixture was heated at 60 °C for 60 min.

Metabolites were analyzed using an Agilent 7890B gas chromatography system coupled with an Agilent 7000E triple quadrupole mass detector (Agilent Technologies, Santa Clara, CA, USA). Chromatographic separation was achieved on a DB-5 MS capillary column (30 m × 0.25 mm × 0.5 μm; Agilent Technologies). The temperature program began at 60 °C and was held for 4 min, increased to 240 °C at a rate of 4 °C/min, then to 280 °C at 10 °C/min, and remaining at this final temperature for 7 min. The injection temperature was kept at 250 °C, with samples injected in splitless mode. Helium (99.999% purity) was used as the carrier gas, with a flow rate of 1.0 mL/min. The ion source temperature was set to 230 °C, and the transfer line to 250 °C. Quantification was carried out in multiple reaction monitoring (MRM) mode.

MRM raw data were analyzed with Quant Analysis software (version 12.0) to determine peak areas for each metabolite. Concentrations were determined using calibration curves designed specifically for each compound. Metabolites associated with HIF-1α were quantified and included pyruvic acid, L-lactic acid, alpha-ketoglutaric acid, and succinic acid.

### 2.6. Histological Studies

The lungs and heart were harvested and histologically examined. For histological analysis, one sample was taken from the most affected region of each of the five lobes [18]. After fixation in 10% neutral buffered formalin, lung tissues were embedded in paraffin and cut into 5 µm sections for histopathological evaluation using hematoxylin and eosin (H&E). Then, immunohistochemical staining was performed with the Ventana BenchMark ULTRA autostainer to ensure consistency. Before staining, tissue samples were subjected to antigen retrieval by heating in a pressure cooker at 125 °C for 30 min with 0.01 M sodium citrate (pH 6.2). The samples were then washed in phosphate-buffered saline and loaded into the autostainer following the manufacturer’s instructions. The primary antibody used for immunohistochemistry was HIF-1α (#MA1-16504, Thermo Fisher Scientific, Waltham, MA, USA). The staining was visualized using Roche Diagnostics’ OptiView DAB IHC Detection Kit. To evaluate the antibodies’ binding abilities, we used positive and negative controls. The positive control had strong staining, whereas the negative control had no significant staining.

A pathologist, who was blind to the experimental protocol and sampling region, conducted the quantitative analysis using light microscopy. Each sample was examined in both low- and high-power fields. Each block was divided into at least four sections, with 20 fields randomly chosen and assessed for each. Histological scoring was blinded and based on six parameters: intra-alveolar edema, hyaline membrane formation, hemorrhage, recruitment of neutrophils into the air spaces, focal alveolar collapse or consolidation, and epithelial desquamation/necrosis of airways or alveoli. The semi-quantitative evaluation was carried out using the following scale: 0 = absent, 1 = mild, 2 = moderate, and 3 = prominent for each parameter [19].

### 2.7. Statistical Analysis

Statistical analyses were carried out with GraphPad Prism 9.0.0 (GraphPad Software, San Diego, CA, USA). Continuous data are presented as mean ± standard deviation. For the porcine ARDS model with repeated measurements, we used repeated measures of one-way ANOVA followed by Dunnett’s multiple comparisons test to detect differences between different time points compared to the baseline. This approach considers the within-subject design of the experiment and compares each time point to the control (baseline) condition. *p* values < 0.05 were considered statistically significant. Data are visualized using grouped tables and graphs that display individual data points as well as summary statistics.

## 3. Results

**Figure 2:**
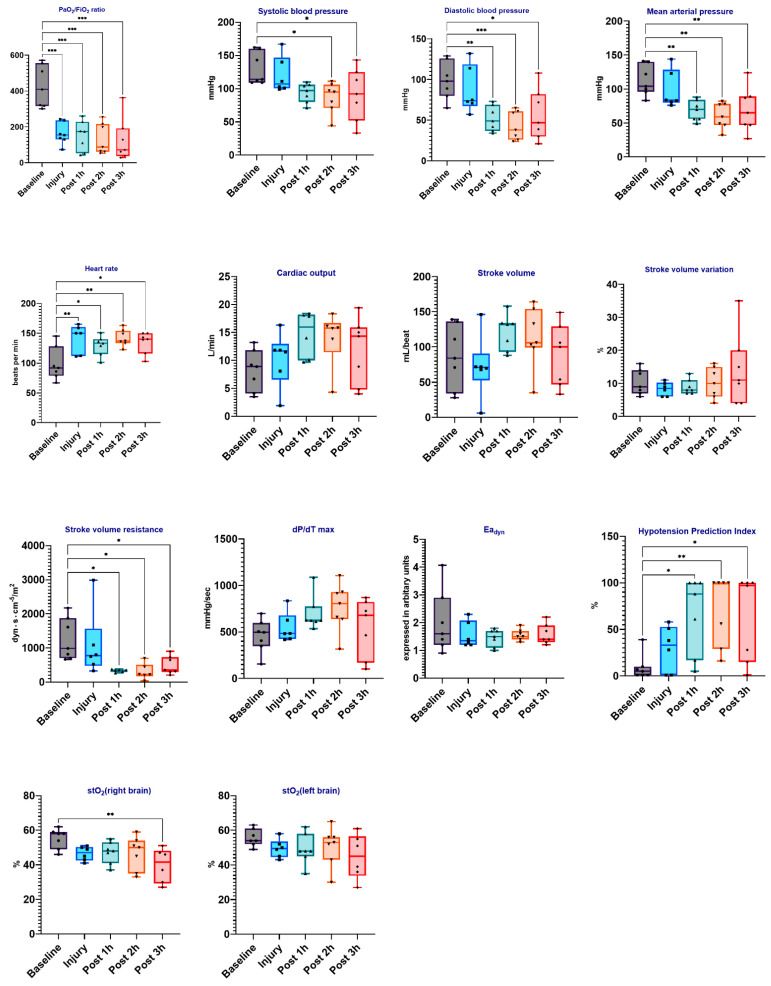
Hemodynamic changes in LPS-induced porcine ARDS model. Box plots depict the distribution of hemodynamic variables (e.g., MAP: mean arterial pressure, SBP: systolic blood pressure, etc.) in the LPS-induced ARDS model at baseline, injury, and various time points post-injury (1 h, 2 h, and 3 h). The data were analyzed with repeated measures of ANOVA. Statistical significance is indicated as follows: * for *p* < 0.05, ** for *p* < 0.01, *** for *p* < 0.001.

In total, eight pigs were used. During the experiments, the animals maintained hemodynamic stability. The HemoSphere platform, which includes the Acumen IQ sensor and the ForeSight Elite sensor, measures hemodynamic parameters such as dP/dt max, Eadyn, HPI, and StO_2_. Figure 2 summarizes the hemodynamic data.

The results of this preliminary study revealed that all animals had ARDS-related hypotension. The MAP was significantly lower following LPS-induced injury, as indicated by the significant effect (vs. baseline, *p* = 0.009). The median MAP at baseline was 112.3 ± 22.5 mmHg and 69.6 ± 32.8 mmHg after 3 h of injury. The median HR was higher at 134.4 beats/min after 3 h of injury versus 98.9 beats/min at baseline (vs. baseline, *p* = 0.010). Although CO was higher in the LPS-induced ARDS period, the difference in CO between the baseline and each time point did not differ significantly. The SVR decreased significantly after the LPS injury compared to the baseline point (vs. baseline, *p* = 0.041), from a median SVR of 1262 mL/min/m^2^ at the baseline point to 439.3 mL/min/m^2^ after 3 h of injury. ARDS with concurrent hypotension/distributive shock resulted in a significant increase in HPI (*p* = 0.024). The SV, SVV, dP/dt max, and Eadyn values were not significantly different between groups. Interestingly, brain StO_2_ levels were significantly lower 3 h after injury compared to baseline (right: from 55.14 ± 5.79 to 39.67 ± 9.83, *p* = 0.003).

**Figure 3:**
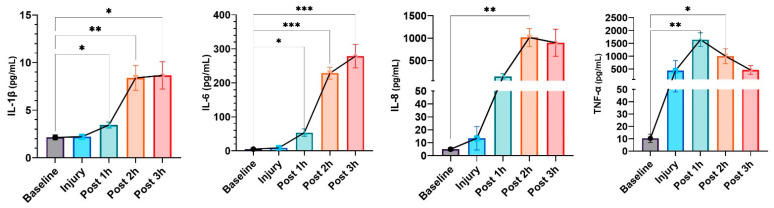
ELISA detection of serum cytokines in an LPS-induced porcine ARDS model. Results are presented as mean ± standard deviation. The data were analyzed with repeated measures of ANOVA. Statistical significance is indicated as follows: * for *p* < 0.05, ** for *p* < 0.01, *** for *p* < 0.001.

Increased levels of cytokines such as IL-1β, IL-6, IL-8, and TNF-α were found to be associated with the progression of hypotension (Figure 3). Specifically, IL-1β levels significantly increased from baseline to 3 h post-injury, while IL-6 also peaked at this time. IL-8 levels also increased significantly, particularly between the injury site and 2 h after the injury. 

TNF-α levels significantly increased post-injury, with a slight decrease at 3 h post-injury but still elevated compared to baseline. These findings point to a strong inflammatory response associated with the hypotensive phases after injury. 

**Figure 4:**
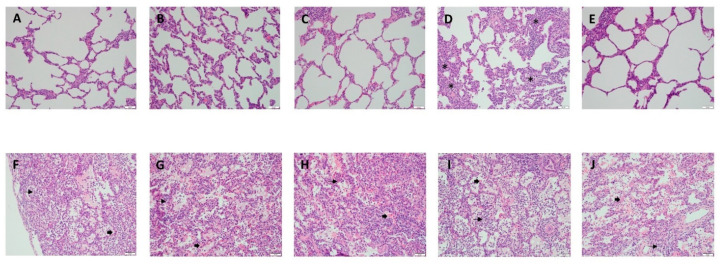
Representative histology of lung injury before and after LPS instillation. Panels (A–E) show the histology of lung lobes prior to LPS instillation. (**A**) RUL (**B**) RML (**C**) RLL (**D**) LUL (**E**) LLL. In each panel, the alveolar sacs show flattened epithelial cells without inflammatory cells, except LUL shows mild hyperplasia of fibroblasts (labelled as *) in the interstitium. Panels (**F**–**J**) show the histology of the lung lobes at 3 h after LPS instillation, revealing significant injury. (**F**) RUL (**G**) RML (**H**) RLL (**I**) LUL (**J**) LLL. Each panel depicts alveolar sacs with hyaline membranes (labelled as arrow heads), intra-alveolar edematous, and acute inflammatory cells such as neutrophil aggregation (labeled as arrows). The scale bars measure 50 μm. RUL, right upper lobe; RML, right middle lobe; RLL, right lower lobe; LUL, left upper lobe; LLL, left lower lobe.

**Figure 5:**
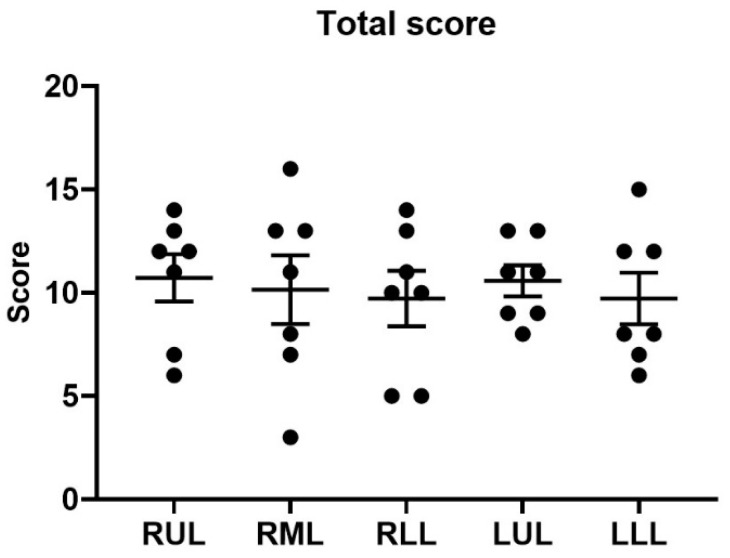
Total lung injury scores across different pulmonary lobes. This figure depicts the distribution of total lung injury scores measured in various pulmonary lobes following LPS instillation. Each dot represents the total injury score for an individual lobe in a single experimental subject, while the horizontal lines represent the mean and standard error for each group. This visualization highlights the severity of lung injury across different lobes in response to LPS-induced injury. RUL, right upper lobe; RML, right middle lobe; RLL, right lower lobe; LUL, left upper lobe; LLL, left lower lobe.

Histological examination of lung tissues from our LPS-induced model revealed uniform lung injury throughout all pulmonary lobes, in stark contrast to the minimal changes observed in the non-injured control experiments (Figure 4). The intra-alveolar edema scores show mild to moderate fluid accumulation within the alveoli, with the right lower lobe and left upper lobe (LUL) having the highest average scores (1.57 ± 0.98 and 1.57 ± 0.79). The presence of hyaline membranes, which indicate acute lung injury, was moderately noted throughout all lobes. The right middle lobe had a higher hemorrhage rate (1.86 ± 1.07), indicating more severe injury compared to other areas. Neutrophils infiltration, an indicator of an inflammatory response, was most severe in the RUL (2.86 ± 0.38). Collapse or consolidation, which may indicate chronic or severe underlying pathology, was moderate but highest in the left lower lobe with a score of 2.0 ± 0.82. The LUL exhibited significant epithelial damage (2.43 ± 0.53), indicating cellular injury (Table 1, Figure 5).

**Figure 6:**
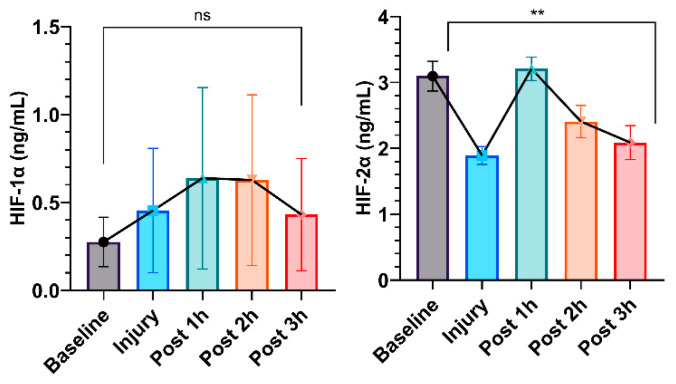
ELISA detection of serum HIF-1α and HIF-2α in LPS-induced porcine ARDS model. Results are presented as mean ± standard deviation. The data were analyzed with repeated measures of ANOVA. Statistical significance is expressed as follows: ** for *p* < 0.01, and “ns” means not significant.

Serum HIF-1α protein levels increased slightly, with a median of 0.28 ng/mL (interquartile range [IQR], 0.09–0.41 ng/mL) compared to 0.43 ng/mL (IQR, 0.08–0.27 ng/mL; *p* = 0.99). Conversely, serum HIF-2α protein levels significantly decreased after 3 h of ARDS injury (median, 3.09 ng/mL [IQR, 2.74–3.23 ng/mL] vs. 2.09 ng/mL [IQR, 1.60–2.25 ng/mL]; *p* = 0.008), as shown in Figure 6.

**Figure 7:**
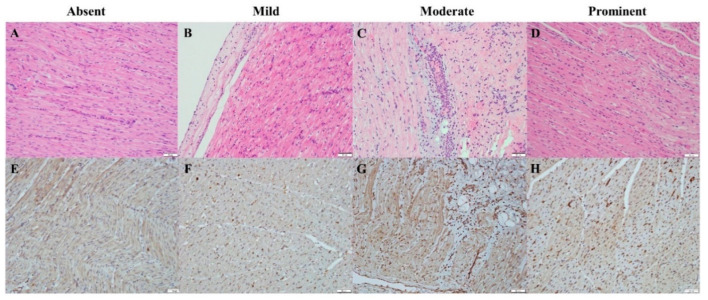
Representative histology of the heart with an immunohistochemical stain for HIF-1α expressing hearts under a 40× objective. (**A**–**D**) hematoxylin and eosin stains, (**E**–**H**) HIF-1α immunohistochemical stains. The scale bars measure 50 μm.

Figure 7 shows the results of H&E and immunohistochemical staining in the heart: (A) and (E) depict the baseline conditions; (B) and (F) show light staining; (C) and (G) show moderate staining, and (D) and (H) show prominent staining. The immunohistochemical results show a higher immunoscore for HIF-1α post-injury compared to baseline, consistent with the ELISA findings.

**Figure 8:**
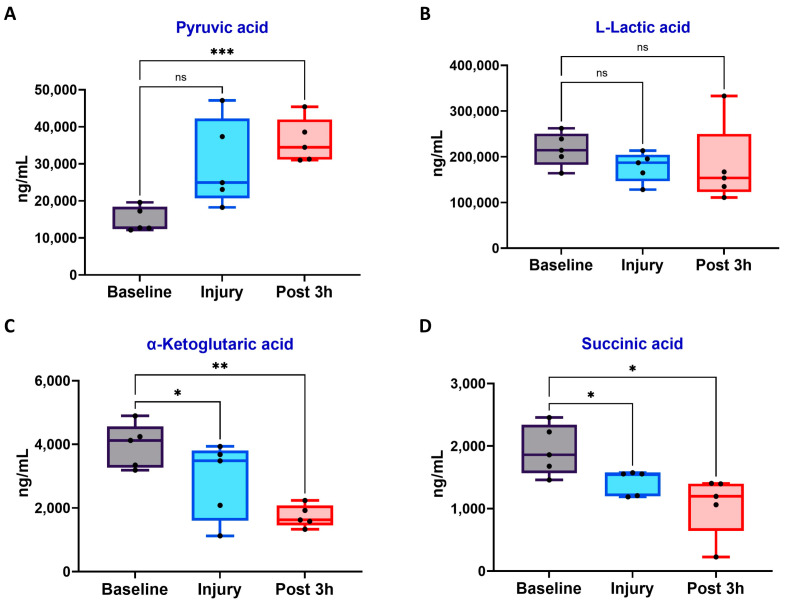
Metabolic changes in LPS-induced ARDS models. HIF-1α-related metabolites levels in porcine blood samples at baseline, injury (LPS-induced ARDS), and 3 h post-injury. (**A**) Pyruvic acid, (**B**) L-Lactic acid, (**C**) α-Ketoglutaric acid, and (**D**) Succinic acid levels. Data are shown as box plots (n = 5 per group). * *p* < 0.05, ** *p* < 0.01, *** *p* < 0.001, ns: not significant; repeated measures of one-way ANOVA with Tukey’s post-hoc test.

The analysis of HIF-related metabolites revealed significant changes after LPS-induced ARDS (Figure 8). Specifically, pyruvic acid levels increased significantly from baseline to post-injury (*p* < 0.001), indicating HIF-1α-induced metabolic reprogramming as previously described [20]. Although not statistically significant, L-lactic acid showed an increasing trend, suggesting a dynamic balance between HIF-1α-mediated lactate production and subsequent clearance mechanisms [21]. Alpha-ketoglutaric acid (α-KG) levels decreased significantly from baseline to injury (*p* < 0.05), with additional reductions observed three hours post-injury (*p* < 0.01 compared to baseline). Similarly, succinic acid levels significantly decreased from baseline at the injury and three-hour post-injury time points (*p* < 0.05), indicating complex metabolic changes within the tricarboxylic acid (TCA) cycle during acute lung injury.

## 4. Discussion

Our study sought to determine the efficacy of the novel Acumen IQ sensor in predicting arterial hypotension and detecting shock in ARDS. We used the HPI along with parameters such as arterial dP/dt max and Eadyn to assess the onset of shock in ARDS. Furthermore, we investigated the ForeSight Elite sensor’s ability to detect cerebral hypoxia promptly and the role of the HIF-α signaling pathway in the cardiovascular system.

The Surviving Sepsis Campaign establishes the standard of care for managing septic shock. Notably, patients who remain in shock after initial resuscitation for 3 h have higher mortality rates [22]. Given the significant impact of ARDS-induced cardiovascular events on mortality and morbidity, arterial pressure is identified as a critical factor. Therefore, maintaining cardiovascular function within the physiologically optimal range for tissue perfusion is critical. The Acumen HPI software, which uses artificial intelligence and machine learning, predicts impending hypotension, defined as a MAP of less than 65 mmHg, for at least one minute [23]. A higher HPI score suggests an increased risk of hypotension. Because the HPI parameter is novel and there were few publications on hemodynamic evaluation in ARDS, we conducted a study using an LPS-induced ALI porcine model.

We discovered that in animals with invasively ventilated ARDS and concurrent hypotension, HPI and HR increased, whereas MAP and SVR decreased significantly. The ForeSight Elite sensor, which was attached to both the right and left forehead, monitored tissue oxygenation and microcirculation health [13]. StO_2_ levels measured with this sensor fell significantly from the baseline, indicating poor microcirculation. Factors such as arterial CO_2_ levels, cardiac output, arterial blood pressure, hemoglobin concentration, and anesthesia depth impact StO_2_ values [24]. Therefore, while StO_2_ values may not accurately reflect cerebral oxygenation status, they can be used to track the trends in StO_2_ changes. Reduced blood pressure and cardiac output can cause cerebral desaturation, resulting in various neurological complications [25].

LPS activates HIF-1α via a Toll-like receptor 4-dependent mechanism, which leads to the activation of cytokines, manifestation of symptoms, and inflammation. HIF-1α modulation is a potential therapeutic target for chronic inflammatory disorders, including sepsis, as it plays a crucial role in hypoxia-induced inducible nitric oxide synthase expression [26,27]. Interestingly, HIF-1α levels in human skin tissue show an inverse correlation with systemic blood pressure. On the contrary, hypertensive individuals typically have higher HIF-2α levels [28]. Intermittent hypoxia also causes increased sympathetic nerve activity and hypertension, pointing to a link between HIF, hypoxia, and blood pressure regulation [29]. These observations highlight the distinct and significant effects of the two HIF-α isoforms on vascular tone and blood pressure, especially in ARDS where hypoxia is a common challenge. 

Our data show a decrease in serum HIF-2α levels during ARDS-associated hypotension. In ischemic conditions, HIF-1α promotes cardioprotection [30], while HIF-2α’s role in myocardial disease is more complex. Inhibiting HIF-2α can reverse right heart failure and pulmonary arterial hypertension [31], while deleting cardiomyocyte-specific HIF-2α can worsen myocardial ischemia-reperfusion injury [32]. These findings highlight the complex roles of HIF-1α and HIF-2α in cardiovascular dysfunction during ARDS, potentially contributing to both the development of ARDS and shock. However, Sato and Takeda suggest that further research into HIF-α signaling can shed light on the molecular pathogenesis of cardiovascular diseases [28]. These include the effects of HIFs on atherosclerosis, cardiovascular development, heart failure, hypertension, and stroke [29].

ARDS-induced hypoxia causes metabolic shifts, including anaerobic glycolysis and disruptions in the TCA cycle, indicating HIF-1α activation. Specifically, the accumulation of pyruvate supports HIF-1α-mediated metabolic reprogramming [20]. This pattern may indicate a balance between increased lactate production due to HIF-1α-induced upregulation of lactate dehydrogenase A (LDHA) and its subsequent clearance [21]. Additionally, a decrease in α-KG may reduce the activity of prolyl hydroxylases, potentially stabilizing HIF-1α and intensifying the hypoxic response [33]. The unexpected decrease in succinic acid levels suggests a more complex metabolic adaptation unique to our ARDS model, possibly due to altered TCA cycle dynamics or mitochondrial dysfunction. These findings demonstrate the rapid and significant metabolic reprogramming occurring in ARDS, outlining the pivotal role of HIF-1α in facilitating cellular adaptation to hypoxic conditions during acute lung injuries.

The critical impact of ARDS-associated arterial hypotension on patient outcomes necessitates a shift from reactive to proactive treatment strategies, with predictors such as HPI [34]. Accurate, continuous hemodynamic monitoring is critical, and while the FloTrac system provides reliable cardiac output measurements [35], insufficient blood flow can cause myocardial hypoxia, dysfunction, and necrosis, implying that an innovative and intriguing approach with HPI monitoring can assist clinicians in better managing hypotension [26]. However, additional clinical validation is required to confirm HPI’s efficacy in improving patient outcomes. Furthermore, early detection of arterial hypotension hinges on accurately identifying the pathophysiological mechanisms that cause low blood pressure and selecting optimal treatment strategies [36]. This study used the Acumen IQ sensor and the ForeSight Elite sensor, which may not apply to other types of monitors, but it can be viewed as a pilot study, particularly since the prediction of ARDS-complicated hypotension was based on the Acumen IQ sensor with HPI value. More research with a larger sample size and a diverse range of monitoring devices is needed to confirm our findings. Furthermore, it is critical to assess the impact of ARDS-induced hypotension by investigating its relationship with serum HIF protein levels and cerebral StO_2_ levels, to gain a comprehensive understanding of this complex issue. 

## 5. Conclusions

In the ICU, managing hypotensive events remains a significant challenge, especially for patients with ARDS. The HPI has proven to be extremely effective at predicting these events, highlighting the importance of advanced monitoring technologies in the ICU setting. Given the complex interplay of HIF signaling, metabolic reprogramming, and cardiovascular responses observed in ARDS, additional research is required to understand the underlying pathophysiology of shock in these patients. Such insights are critical for the development of personalized hemodynamic therapies that not only address fluid management and respiratory support but also incorporate biomarker evaluations to tailor treatments to individual patient needs.

## Figures and Tables

**Table 1:** Histologic examination.

Parameter	RUL	RML	RLL	LUL	LLL
Intra-alveolar edema	1.14 ± 0.69	0.86 ± 0.69	1.57 ± 0.98	1.57 ± 0.79	1.29 ± 0.95
Hyaline membranes	1.57 ± 0.79	1.71 ± 0.95	1.57 ± 1.13	1.57 ± 0.53	1.43 ± 0.98
Hemorrhage	1.0 ± 0.58	1.86 ± 1.07	1.29 ± 0.49	1.14 ± 0.69	1.14 ± 1.22
Neutrophils infiltration	2.86 ± 0.38	2.14 ± 1.07	2.14 ± 0.89	2.57 ± 0.79	2.29 ± 0.76
Collapse or consolidation	1.86 ± 1.35	1.86 ± 1.07	1.57 ± 0.79	1.29 ± 0.76	2.0 ± 0.82
Epithelial damage	2.29 ± 0.76	1.71 ± 1.11	1.57 ± 0.98	2.43 ± 0.53	1.57 ± 1.13

Definition of abbreviations: RUL, right upper lobe; RML, right middle lobe; RLL, right lower lobe; LUL, left upper lobe; LLL, left lower lobe.

## Data Availability

The datasets used and/or analyzed in the current study are available from the corresponding author upon reasonable request due to ethical considerations and the presence of unpublished data.

## Abbreviations

ARDS, Acute respiratory distress syndrome; PEEP, positive end-expiratory pressure; CO, cardiac output; RV, right ventricular; SVR, systemic vascular resistance; ICU, intensive care unit; SVV, stroke volume variation; HIFs, hypoxia-inducible transcription factors; HPI, hypotension prediction index; dP/dt max, arterial maximum arterial pressure rise; EtCO_2_, end-tidal CO_2_ pressure; LPS, lipopolysaccharides; MAP, mean arterial pressure; HR, heart rate; SV, stroke volume; SVV, stroke volume variation; SVR, systemic vascular resistance; dP/dt, systolic pressure increase divided by duration of systole; Ea_dyn_, dynamic arterial elastance; StO_2_, tissue oxygen saturation.
